# Supplementary material for: TorchGWAS 1.0: GPU-accelerated GWAS at scale
Source: Bioinform Adv. 2026 Oct 13;6(1):vbag310. doi: 10.1093/bioadv/vbag310 (PMC13686528; doi:10.1093/bioadv/vbag310)
Supplement: Supplementary file 1 [file vbag310_supplementary_data.docx]

# Supplementary Information

# TorchGWAS 1.0: GPU-accelerated GWAS at scale

Xingzhong Zhao^1,2†^, Ziqian Xie^1,2†^, Sheikh Muhammad Saiful Islam^1,2^, Tian Xia^1,2^, Cheng Chen^1^, Degui Zhi^1,2*^

^1^Department of Bioinformatics and Systems Medicine, McWilliams School of Biomedical Informatics, The University of Texas Health Science Center at Houston, Houston, Texas, USA

^2^ Department of Biomedical Informatics and Data Science, Yale School of Medicine, New Haven, Connecticut 06510, USA

† Xingzhong Zhao and Ziqian Xie contributed equally to this work.

*Corresponding author. Degui Zhi, Department of Biomedical Informatics and Data Science, Yale School of Medicine, New Haven, Connecticut 06510, USA

E-mail: [Degui.Zhi@yale.edu](mailto:Degui.Zhi@yale.edu)

# Supplementary Methods

## S1. Statistical model and numerical evaluation

### S1.1 Covariate-adjusted fixed-effect OLS

For a cohort of *N* aligned samples, one quantitative phenotype vector *y* and one tested dosage vector *g*, TorchGWAS fits the fixed-effect model

$$y = \alpha1 + C\gamma+ g\beta+ \varepsilon$$

Here, *y* and *g* are *N*-vectors. *1* is the *N*-vector of ones. *C* is the *N* × *c* matrix of *c* numeric non-intercept covariates. *α* is the intercept. *γ* is the covariate-coefficient vector. *β* is the tested genotype effect, and *ε* is the residual vector. Samples are aligned before analysis, covariates must be finite, and categorical covariates must be represented by numeric indicator columns. TorchGWAS does not include a genomic relationship matrix or another random effect. Studies retaining related individuals require an appropriate mixed-model method.

Phenotypes and covariates are centered. A rank-revealing singular value decomposition of the centered covariate matrix yields an N × q orthonormal basis Q, where q is the numerical covariate rank after redundant columns are removed. The numerical rank is determined in float64 with numpy.linalg.svd. After column centering and standardization, a singular value is retained only when

$$s_{j}>tol, tol=max\left( N,c \right) \epsilon_{64} s_{\max}, \epsilon_{64}=2.220446049250313\times{10}^{-16}$$

The numerical constant in this expression is float64 machine precision, and the largest-singular-value factor is taken from the same decomposition. The retained orthonormal basis is then cast back to the requested analysis precision. Each phenotype is projected off Q and divided by its post-projection standard deviation across the analyzed samples, computed with denominator N. Here, y* denotes this processed phenotype. For a centered genotype vector g, the sufficient statistics are

$$u = gᵀy*,\quad\quad v = gᵀg - \left\| Qᵀg \right\|^{2}$$

$$\hat{\beta} = u/v,\quad\quad\mathrm{RSS} = \|y*\|^{2} - u^{2}/v$$

$$\mathrm{SE}\left( \hat{\beta} \right)= \sqrt{\left[ \frac{\frac{RSS}{d}}{v} \right]},\quad\quad t =\frac{\hat{\beta}}{\mathrm{SE}\left( \hat{\beta} \right)},\quad\quad d = N - q - 2$$

In these equations, u is the genotype–phenotype cross-product numerator, v is the residual genotype sum of squares, RSS is the regression residual sum of squares, SE is the coefficient standard error, t is the signed Student statistic and d is its residual degrees of freedom. N is the number of samples and q is the numerical covariate rank. Because q excludes the intercept, the subtraction of 2 in d = N − q − 2 accounts for the intercept and the tested genotype. The products with Q evaluate genotype residualization without materializing a residualized genotype matrix. For a block of B variants and K phenotypes, where B is the number of variants in the block and K is the number of phenotypes, the genotype–phenotype products produce all B × K numerators in one batched operation, followed by small covariate-projection products and element-wise regression calculations.

### S1.2 Missing genotype and phenotype values

For a genotype or phenotype column containing missing entries, the column mean is calculated from observed values and missing entries contribute zero after centering. This removes missing cells from the batched dot products without constructing a different row-subset design matrix for every association. It is algebraically equivalent to filling a missing cell with that column’s observed mean for the cross-products. It is not a per-pair complete-case refit, and the covariate basis is not re-estimated for each missingness pattern.

Observed counts are carried into the uncertainty calculation. Let $n_{g}$be the number of samples with an observed genotype for the tested variant, and let $n_{y}$ be the number with an observed value for the tested phenotype. With N denoting the number of aligned samples and q the numerical covariate rank, define

$$d_{0}=N-q-2, d_{g}=n_{g}-q-2, d_{y}=n_{y}-q-2$$

Genotype missingness therefore changes the variant-level residual degrees of freedom. Phenotype missingness rescales the statistic by

$$\sqrt{d_{y}/d_{0}}$$

In the reference in-memory path, the two-sided Student t tail uses the combined effective degrees of freedom

$$d_{\mathrm{eff}}=\frac{d_{g}d_{y}}{d_{0}}$$

Thus, uncertainty can vary with both the variant and phenotype even though the shared covariate basis and matrix products use the aligned cohort.

The current dense streaming format stores both t and −log_10_*P* for each association and records phenotype-level degrees of freedom in its manifest. The −log_10_*P* value is evaluated during the scan using the applicable effective degrees of freedom, so variant-specific tails are retained even when genotype observation counts vary. Reconstructing those tails later from t alone would still require the observed genotype counts. This distinction does not affect the complete-genotype concordance experiments reported here.

Accordingly, under missingness, TorchGWAS uses a shared-basis batched formulation rather than refitting the full covariate design separately for each variant–phenotype pair.

### S1.3 Tail probabilities, scale and allele direction

To avoid loss of ranking when an extreme Student *t* tail underflows in ordinary floating-point survival functions, TorchGWAS evaluates −log10*P* from the regularized incomplete-beta representation

$$P\left( \left| T \right|\geq\left| t \right| \right)=I_{x}\left( d/2,1/2 \right), x=\frac{d}{\left( d+t^{2} \right)}$$

where *T* is a Student random variable with *d* degrees of freedom and *I*x(*a*, *b*) is the regularized incomplete beta function. The expression is evaluated in log space with PyTorch tensor operations, and the final exponential is not formed for −log10*P*. Reported *P* values are two-sided.

The phenotype is expressed in residual-phenotype standard-deviation units, while genotype remains on the input dosage scale. The reported β is therefore the change in residual-phenotype standard deviations per copy of the declared effect allele. BED input counts BIM A2, PGEN input counts ALT, and BGEN input counts the second allele in the variant record. The effect allele is stored with the results, and cross-format or cross-tool comparisons are allele-aligned before signed statistics are compared.

Association arithmetic can use float32 or float64. A tested variant is valid only when its observed genotypes are nonconstant, its residual genotype sum of squares is finite and strictly greater than 10⁻¹², and its residual degrees of freedom are positive. The 10⁻¹² cutoff is absolute rather than scale-relative. Float64 is intended mainly for validation or hardware with suitable double-precision performance. The production benchmarks used float32 unless otherwise stated.

## S2. Direct-format streaming pipeline

### S2.1 Native-format block production

TorchGWAS accepts BED, PGEN files containing hard calls or dosages, and BGEN as primary genotype inputs. Let *M* be the total number of variants and *B* the selected variant chunk size. The scan contains ⌈*M*/*B*⌉ ordered blocks. The final block contains the remainder when *M* is not divisible by *B*. Format-specific readers retain BED, PGEN and BGEN records in their compact source representation until the block is needed. Each block is read and decoded directly into reusable host or device buffers, so the pipeline never materializes a complete *N* × *M* genotype matrix. At the implementation level, BED and native hard-call PGEN readers use offset-based os.preadv calls, and BGEN uses os.pread, allowing workers to read assigned byte ranges without sharing a mutable file position. PGEN dosage and unsupported record modes use pgenlib.PgenReader.

### S2.2 Bounded overlapped execution

Each variant block moves through a bounded pipeline: native-format read/decode, host buffering, host-to-device transfer, GPU association, device-to-host result collection and background output writing. Different blocks can occupy different stages at the same time. Reusable rings bound memory use, CUDA events enforce dependencies and buffer ownership, ordered handoff preserves variant order, and full downstream queues propagate backpressure to earlier stages. The genotype formats differ mainly in the read/decode front end and converge on the same ordered association and output stages.

TorchGWAS uses bounded rings of reusable buffers rather than allocating new memory for every variant block. In the general dosage and PGEN paths, the pipeline maintains D page-locked (pinned) host buffers, D GPU input buffers and D pinned result buffers, where D is the configured prefetch depth. Two queues record which host buffers are free and which contain data ready for transfer. Up to W = min (D, $n_{worker}$, J) reader or decoder tasks can fill free buffers, where n_worker is the configured worker count and J is the number of remaining blocks. Blocks are returned in variant order even if worker tasks finish out of order. When no buffer is free, reading pauses until a downstream stage releases one, which prevents an unbounded backlog and keeps memory use fixed.

Input preparation differs by genotype format. BED reader threads read packed two-bit rows by file offset directly into pinned ring buffers, without first decoding the genotypes on the CPU. PGEN workers decode hard calls or dosages directly into pinned buffers, and each worker owns an independent PGEN reader. For BGEN GPU decoding, file reads and GPU decoding use separate bounded worker pools. Each decoding lane has its own CUDA stream, decoder handle and working buffers, so a later block can be read while an earlier block is being decoded. The decoded blocks are still passed to association testing in variant order.

GPU work uses three CUDA streams: one for association calculations, one for copying genotype blocks to the GPU and one for copying results back to host memory. In the implementation, these are persistent torch.cuda.Stream objects, and each stage submits work inside a torch.cuda.stream() context. Transfers between pinned host buffers and GPU tensors are requested with non_blocking=True. Dependencies are represented by torch.cuda.Event objects: the genotype-transfer stream records an event after the input copy, the calculation stream waits for that event before using the block and records a second event when association testing finishes, and the result stream waits for the calculation event before copying β, t, −log10P, variant status and residual degrees of freedom back to the corresponding pinned result buffer. It then records a final event marking completion of the result copy.

Events are recorded separately for each ring position. An input position is not overwritten until the previous calculation using it has finished, and a result position is not reused until its previous result has been collected. Calls to record_stream() tell PyTorch which stream still uses each tensor, preventing the underlying storage from being recycled too early. A bounded CPU worker pool waits for completed result events, converts the pinned tensors into ordinary owned arrays, records exclusion counts and performs any requested result processing while the GPU works on later blocks. If the pending-result queue becomes full, the main loop collects the oldest result before accepting another block. This preserves variant order and carries backpressure from output processing to the earlier pipeline stages.

The exact ring geometry is format-specific. The general dosage path uses the configured prefetch depth for its input, GPU and result rings. The packed BED path uses a host input ring with at least three positions and normally two more positions than the number of BED reader threads, a four-position packed GPU-input ring and a separately sized result ring limited by CPU result concurrency and a 1-GiB pinned-memory budget. The same buffer-ownership and event rules apply to each path. Setup, ordered handoff, waits caused by full rings and final synchronization are not asynchronous. During steady-state execution, however, reading, transfer, calculation, result collection and writing can operate on different blocks at the same time. End-to-end wall time therefore includes pipeline fill, the slowest steady-state service, pipeline drain and final output completion rather than the sum of isolated stage times.

### S2.3 Chunk selection and memory bounds

When the user does not specify the number of variants in each streamed block, TorchGWAS queries free device memory and estimates the memory required by the phenotype and covariate design, genotype-transfer buffers, decoded genotype workspace, covariate-projection products and result buffers. The query uses torch.cuda.mem_get_info(). In float32, the phenotype and covariate design requires approximately four bytes multiplied by the sample count and by the sum of the phenotype count, covariate rank and one intercept column. The genotype and result terms increase with the number of variants per block. The transfer buffers also increase with pipeline depth and with the number of bytes transferred for each variant in the input format. The automatic rule chooses the largest permitted block, up to 4,096 variants, whose estimated buffers fit within 85% of reported free device memory. For framed formats, it rounds down to a frame-aligned size when at least one complete frame fits. If even the smallest candidate does not satisfy that budget, it returns the minimum block of 128 variants and the subsequent allocation provides the definitive feasibility check.

Because blocks are released and ring slots are reused, the total number of variants changes the number of iterations but not the steady-state ring size. Peak device memory therefore depends mainly on the number of samples, the number of phenotypes held on the GPU at one time, the covariate rank, the number of variants per block, numerical precision, compact genotype representation and pipeline depth. The phenotype matrix remains on the GPU throughout a scan. A phenotype panel that does not fit must currently be divided into non-overlapping groups.

### S2.4 Dense binary output

The default dense output writes three float32 arrays: beta.f32, tstat.f32 and neglog10p.f32. Together they have an exact payload of 12MK bytes before metadata, where M is the number of variants and K is the number of phenotypes. Screening-only output omits beta.f32 and writes 8MK bytes. Each output array has a background writer thread and a bounded ring of reusable output blocks. The block size is selected between 1 and 16 MiB from the first result chunk, and the default queue depth is three. A complete contiguous result block can be passed directly to its writer when the scan no longer needs it. Smaller blocks, or blocks still owned by the scan, are copied into reusable staging buffers.

The writers are Python threading.Thread workers fed by bounded queues, and their drain loops issue os.write calls while later variant blocks are transferred and evaluated on the GPU. On Linux systems that provide sync_file_range, each completed 64-MiB range is submitted to the operating system for background writeback during the scan. Older completed ranges are then waited on and removed from the page cache, which limits accumulated unwritten data and shortens the final synchronization to storage. If storage cannot keep pace, the bounded output rings fill and pause the scan-side append operation instead of allowing memory use to grow without limit. At the end of the scan, all writer queues are drained, outstanding writeback is completed and each output file is synchronized to durable storage with os.fsync before the manifest and sidecars are finalized.

### S2.5 Supported hardware and measured memory

Accelerated execution requires a CUDA-capable NVIDIA GPU and a compatible PyTorch/CUDA installation. The tested systems had compute capability 7.5 or newer. Compute capability 6.1 should also be supported when the installed software stack and compiled kernels include that architecture. In full-genome BED runs with 35,365 subjects, peak GPU allocation was 0.8–1.9 GB across 1–2,048 phenotypes. Other formats can require larger staging buffers, as reported in Supplementary Table S5.

## S3. Statistical validation experiments

### S3.1 Direct OLS and PLINK 2.0 concordance

The direct-OLS regression test used 156 complete-case samples, 48 variants, four quantitative phenotypes and three genotype-derived principal components, yielding 192 variant–phenotype tests and 151 residual degrees of freedom. Direct full OLS included an intercept, the three principal components and the tested genotype. TorchGWAS was evaluated in float64 and float32. PLINK 2.0 used the same samples, phenotypes and covariates. Effect alleles and phenotype scale were aligned before comparison.

A separate PLINK 2.0 audit evaluated TorchGWAS in float32 mode across three validation datasets: the repository toy data (64 samples, 12 variants, three phenotypes and four covariates), a structured synthetic dataset (2,000 samples, 10,000 variants, 10 phenotypes and five covariates), and a real-genotype dataset (12,405 samples, 100,000 variants, four simulated phenotypes and five covariates). Variant IDs and allele metadata were harmonized rather than matched by row position. The final comparison contained 500,036 matched t statistics.

### S3.2 Structured-population null simulation

Real genotypes from the available replication subset preserved linkage disequilibrium and population structure. Each analysis used 500 individuals, 200 variants and three genotype principal components. Four replicates of 1,000 null phenotypes were generated at each of four proportions of phenotype variance explained by the population-structure component (0%, 5%, 20% and 40%), producing 800,000 tests per setting and 3.2 million tests overall.

The evaluated methods were TorchGWAS, PLINK 2.0 OLS, a no-PC fixed-effect model and the original phenotype-only residualization formula. Empirical rejection rates were recorded at 0.05, 10⁻³ and 10⁻⁵. Genomic inflation was summarized by the genomic inflation factor λGC. Because tests within a setting reuse genotypes and variants, the rejection rates are reported descriptively rather than with binomial confidence intervals. The experiment was powered for the 0.05 and 10⁻³ thresholds. The sparse 10⁻⁵ counts were treated descriptively.

### S3.3 Structured-population power simulation

The power study used 5,000 individuals, principal-component variance levels of 0%, 5%, 20% and 40%, causal minor-allele-frequency targets near 0.01, 0.05, 0.20 and 0.40, standardized effect sizes of 0.05–0.80, weak/moderate/strong causal-variant–PC correlation and one- or three-causal-variant architectures. These factors produced 480 conditions, each with 20 replicates. Power was the proportion detecting the causal variant, or at least one causal variant in the multi-causal architecture, at P < 5 × 10⁻⁸. Effect sizes in this simulation were standardized by genotype SD; this parameterization differs from the per-dosage-copy $\beta$reported by TorchGWAS.

## S4. Benchmark and runtime-predictor design

### S4.1 End-to-end timing boundary and full-genome formats

The modeled timing boundary began after the shell, Python interpreter, package imports and benchmark environment were ready, but before CUDA context initialization or study-file access, and ended after the requested result files had been fully written and synchronized to storage with os.fsync. Environment construction and process-launch service are outside the model. The retained cold analytical start includes CUDA context creation, first-use library initialization, cold metadata and genotype reads, parsing and quality control, covariate-basis construction, pinned-buffer allocation, GPU design construction, genotype input and decoding, association testing, result transfer, output writing and final sidecars. Each measured TorchGWAS run used a fresh analytical invocation, and the stated cold runs evicted both genotype and metadata file pages from the operating-system page cache before timing.

The full-genome format experiment used one NVIDIA H100 80-GB GPU, a 48-core Intel Xeon Gold 6442Y host, N = 35,365 samples, M = 8,931,083 variants, K = 512 phenotypes and 27 covariates. Measured sequential disk read and write rates were 5.98 and 1.49 GB / s. Genotype pages and metadata caches were cold, formats were interleaved across two rounds, and each run wrote 36.7 GB of β+t output. Peak resident host memory and peak GPU allocation/reservation were collected from the same runs.

A hard-call PGEN stage profile with 512 phenotypes (K = 512) recorded 16.84 s of fetch/decode service, 9.05 s of GPU association service and 0.81 s of result-transfer service within an 18.98 s scan. The isolated service clocks sum to 26.70 s, which exceeds the scan wall time because stages overlap. This is consistent with the scheduler model of concurrent pipeline stages and identifies fetch/decode as the dominant service in this profile.

### S4.2 Comparator measurements

The comparator experiments used PLINK 2.0 v2.00a7.5LM (10 September 2026), GCTA v1.95.3 for fastGWA-lr, and tensorQTL v1.0.10. PLINK 2.0 benchmarks requested 48 threads. In the tested GCTA executable, the fastGWA-lr association loop was effectively single-threaded. Increasing the requested thread count from 4 to 48 did not materially change runtime. Here, fastGWA-lr refers to GCTA’s fixed-effect linear-regression mode, not its mixed-model modes.

tensorQTL was compared with TorchGWAS in matched no-output H100 runs with N = 8,192, M = 65,536 hard-call PGEN variants and K = 1–32. The CPU-comparator crossover experiments used K = 1 and varied N and M. Quiet-host PLINK 2.0 measurements used fresh processes for both tools, 48 requested PLINK threads and page-cache-preconditioned PGEN input. The fastGWA-lr and PLINK 2.0 transitions are reported as approximate workload scales rather than universal constants because process startup, storage, CPU availability and output formats affect the boundary.

### S4.3 Resource-based runtime predictors

These predictors were used during development to identify bottlenecks and guide pipeline design, then during benchmarking to select comparison workloads and measurements near predicted crossovers. They are source-derived mechanistic models rather than fits to complete GWAS runtimes. The equations are schematic in the sense that they state the dependency structure and source-count equations, while a numerical prediction also requires the selected hardware profile, primitive measurements and implementation traces. Predictor inputs fall into three classes: independently measured hardware or primitive rates, source-counted operations or byte volumes, and explicit modeling assumptions such as uniform encoded work across blocks or ideal-LRU cache traces. Complete GWAS runtimes are used only for validation and measured bracketing near predicted transitions.

Separate predictors are constructed for TorchGWAS, PLINK 2.0 and fastGWA-lr. S4.3.1 gives common timing rules, S4.3.2 gives TorchGWAS setup costs, S4.3.3 gives the finite TorchGWAS scan, S4.3.4 and S4.3.5 give the two CPU comparator models, and S4.3.6 defines the hardware profiles and crossover search. The TorchGWAS implementation supports separate profiles for BED, hard-call PGEN, dosage PGEN and BGEN. The detailed equations below instantiate hard-call PGEN because the matched CPU-comparator experiments use that input.

Throughout this section, $N$, $M$ and $K$ denote the numbers of samples, variants and analyzed phenotypes. $c$ is the number of supplied non-intercept covariates, while $q$ is the numerical rank of the retained covariate basis defined in S1. $K_{\mathrm{file}}$ is the number of phenotype-value columns parsed from the phenotype input file, excluding identifier columns, and can exceed $K$ when only a subset is analyzed. Raw-input parsing, quality-control, copying and SVD terms use $c$. Operations on the retained basis use $q$. In the Figure 2 profile $c=q=8$, so this distinction does not alter the plotted predictions. $B$ denotes the TorchGWAS variant chunk size and $D$ the pipeline depth. $R_{r}$ and $R_{w}$ are sustained input and output rates, while $R_{H2D}$ and $R_{D2H}$ are sustained host-to-device and device-to-host rates. In the predictor notation, lowercase u denotes per-item CPU service, $U$ aggregate CPU service, $H$ logical memory traffic in bytes, $F$ floating-point operation counts and $T$ elapsed service time. I(·) is an indicator function.

#### **S4.3.1 Common timing and resource rules**

For any non-negative work amount $W$ and compatible sustained capacity $R$, service time is $\frac{W}{R}$. Sequentially dependent services are summed. Alternative instruction, arithmetic and memory limits on the same operation are combined by a roofline maximum. Distinct stages may overlap only when the finite execution graph permits them to be active concurrently. Adjacent symbols denote multiplication. Each product below is a count of rows, cells, records, calls or bytes multiplied by the corresponding per-item service or rate. Fixed numeric coefficients are byte widths or source-level operation counts and are explained where they appear.

$$T_{\mathrm{service}}=\frac{W}{R}, T_{\mathrm{sequential}}=\sum_{j} T_{j}, T_{\mathrm{roofline}}=max\left( T_{\mathrm{instruction}},T_{\mathrm{arithmetic}},T_{\mathrm{memory}} \right)$$

A serial CPU service $U$ requires $\frac{U}{f_{\mathrm{CPU}}}$ elapsed seconds. Independently runnable CPU nodes with total service $U_{\mathrm{total}}$ are also constrained by the whole-process floor $\frac{U_{\mathrm{total}}}{C_{\mathrm{CPU}}}$. Other whole-run resource floors are written generically as $\max_{a}\left( W_{a}/R_{a} \right)$, where a indexes a resource, $W_{a}$ is the assigned work and $R_{a}$ is its independently measured sustained capacity. These floors prevent a finite schedule from implying more aggregate throughput than the recorded hardware can provide.

$$T_{\mathrm{serial}}=\frac{U}{f_{\mathrm{CPU}}}, T_{CPU,floor}=\frac{U_{\mathrm{total}}}{C_{\mathrm{CPU}}}, T_{resource,floor}=\max_{a}\left( W_{a}/R_{a} \right)$$

The TorchGWAS cold analytical boundary was decomposed as follows. No environment or process-launch term is present. CUDA context and first-use numerical-library service remain because they occur after the selected boundary. In the next equation, $T_{\mathrm{CUDA}}$ is CUDA context and initial library time, $T_{metadata,IO}$ and $T_{metadata,CPU}$ are metadata input and CPU times, $T_{\mathrm{pin}}$ is pinned-buffer setup time, $T_{\mathrm{design}}$ is GPU design construction time, $T_{first use}$ is the initial call overhead, $T_{scan+close}$ is the finite scan through durable output completion and $T_{\mathrm{sidecar}}$ is final metadata time.

$$T_{\mathrm{Torch}}=T_{\mathrm{CUDA}}+T_{metadata,IO}+T_{metadata,CPU}+T_{\mathrm{pin}}+T_{\mathrm{design}}$$

$$+T_{first use}+T_{scan+close}+T_{\mathrm{sidecar}}$$

#### **S4.3.2 Cold metadata, preprocessing and buffer setup**

For the hard-call PGEN crossover profile, cold metadata service used the measured bytes of the variant, sample, phenotype and covariate files. For PGEN, the index/header bytes were the PGEN file size minus the encoded variant-record payload and were read once before the parallel scan. Genotype-record payload bytes were not charged here because they were assigned block by block to the scan reader. In the next equations, $B_{\mathrm{pvar}}$, $B_{\mathrm{psam}}$, $B_{\mathrm{phenotype}}$ and $B_{\mathrm{covariate}}$ are the measured byte counts of those four metadata files. $B_{\mathrm{PGEN}}$ is the complete PGEN file size, $B_{record payload}$ is its encoded variant-record payload and $B_{\mathrm{index}}$ is the remaining index and header volume. $R_{r}$ in the metadata input equation is the independently measured input rate.

$$B_{\mathrm{metadata}}=B_{\mathrm{pvar}}+B_{\mathrm{psam}}+B_{\mathrm{phenotype}}+B_{\mathrm{covariate}}, B_{\mathrm{index}}=B_{\mathrm{PGEN}}-B_{record payload}$$

$$T_{metadata,IO}=\frac{\left( B_{\mathrm{metadata}}+B_{\mathrm{index}} \right)}{R_{r}}$$

The total metadata CPU service $U_{\mathrm{setup}}$ included row parsing, finite-value checks, phenotype and covariate quality control, numerical-rank determination, initial PGEN-index parsing and the array conversions required by the implementation:

$$U_{\mathrm{setup}}=u_{\mathrm{pvar}}M+u_{\mathrm{psam}}N+u_{pheno,QC}NK+u_{covar,QC}Nc+u_{\mathrm{basis}}\left( Nc^{2}+c^{3} \right)$$

$$+u_{\mathrm{index}}Mn_{\mathrm{index}}+u_{\mathrm{copy}}\left[ 12N\left( K+c \right)+12Nc \right]$$

Each term is a count multiplied by an independently measured unit service. The product $u_{\mathrm{pvar}}$ $M$ is $M$ variant rows times CPU-seconds per variant row, and $u_{\mathrm{psam}}$ $N$ is $N$ sample rows times CPU-seconds per sample row. The product $u_{pheno,QC}$ $NK$ counts $N$ samples $\times$ $K$ phenotype columns, whereas $u_{covar,QC}$ $Nc$ counts $N$ samples $\times$ $c$ supplied covariates. Multiplying each cell count by its corresponding per-cell quality-control service gives CPU-seconds. The basis normalizer $Nc^{2}+c^{3}$ contains $N$ $\times$ $c$² work for operations on the tall $N$-by-$c$ supplied covariate matrix and $c$³ work for the small $c$-by-$c$ factorization and rank decision. The index term $u_{\mathrm{index}}$ $M$ $n_{\mathrm{index}}$ is CPU-seconds per index record $\times$ $M$ records $\times$ the number $n_{\mathrm{index}}$ of complete PGEN-index traversals performed before the worker pipeline begins. In the current hard-call PGEN profile $n_{\mathrm{index}}=2$, one traversal for scope/index inspection and one for initial reader construction. Additional worker-specific index constructions are charged separately by $I\left( i<W \right)Mu_{\mathrm{index}}$ in the finite scan. With four decoder workers and enough chunks, the model therefore charges six complete index traversals in total. In $12N\left( K+c \right)$, $N\left( K+c \right)$ is the number of phenotype and supplied-covariate elements and 12 bytes per element is an 8-byte float64 read plus a 4-byte float32 write. In the additional $12Nc$ term, $Nc$ is the number of supplied covariate elements and 12 bytes is a 4-byte float32 read plus an 8-byte float64 write for the stable rank calculation. Multiplication by $u_{\mathrm{copy}}$ converts these logical bytes to CPU service. Finally, $T_{metadata,CPU}=\frac{U_{\mathrm{setup}}}{f_{\mathrm{CPU}}}$ divides total serial CPU-seconds by the available fraction $f_{\mathrm{CPU}}$ of one logical CPU.

$$T_{metadata,CPU}=\frac{U_{\mathrm{setup}}}{f_{\mathrm{CPU}}}$$

For the following hard-call PGEN buffer calculation, $N$ is the sample count, $B$ is the number of variants in one chunk, $K$ is the phenotype count and $D$ is the number of ring positions. Each pinned input slot contains $NB$ one-byte decoded hard calls. Each result slot contains $4BK$ bytes of float32 coefficients, $4BK$ bytes of float32 $t$ statistics, $8BK$ bytes of float64 $-\log_{10}P$ values before writer-side conversion, $B$ status bytes and $4B$ residual-degrees-of-freedom bytes. The default dense-output pinned-ring allocation is therefore

$$B_{\mathrm{pinned}}=D\left[ NB+\left( 16K+5 \right)B \right], n_{\mathrm{pages}}=ceil\left( B_{\mathrm{pinned}}/4096 \right)$$

$$T_{\mathrm{pin}}=\frac{n_{\mathrm{pages}}u_{pin,CPU}}{f_{\mathrm{CPU}}}+n_{\mathrm{pages}}t_{pin,driver}$$

The product $NB$ is $N$ samples $\times$ $B$ variants $\times$ one byte per decoded hard call in one input slot. In one result slot, $16KB$ consists of $4BK$ coefficient bytes + $4BK$ $t$-statistic bytes + $8BK$ float64 $-\log_{10}P$ bytes, and the additional $5B$ consists of one status byte + four residual-df bytes for each of $B$ variants. Thus one dense slot contains $NB+\left( 16K+5 \right)B$ bytes, and multiplication by $D$ accounts for all $D$ simultaneously allocated ring positions. Dividing this total by 4,096 bytes per operating-system page and rounding upward gives $n_{\mathrm{pages}}$. The product $n_{\mathrm{pages}}$ $u_{pin,CPU}$ is the page count $\times$ measured CPU-seconds per pinned page and is divided by $f_{\mathrm{CPU}}$. The driver term $n_{\mathrm{pages}}$ $t_{pin,driver}$ is the same page count $\times$ measured non-CPU driver time per page. A scan without $-\log_{10}P$ removes its $8BK$-byte component, leaving $\left( 8K+5 \right)B$ result bytes per slot. The configured capacity $B$, rather than the shorter final block, is used because every reusable slot is allocated at full capacity. In the pinned-buffer time, $f_{\mathrm{CPU}}$ is the available fraction of one logical CPU for serial work, $u_{pin,CPU}$ is the measured CPU service for pinning one page and $t_{pin,driver}$ is the measured non-CPU driver time for one page.

GPU design construction is anchored by one directly measured small setup call with $N_{0}=32$ samples, $c_{0}=q_{0}=8$ and $K_{0}=1$ phenotype. These are the dimensions of the timing primitive, not default study dimensions or fitted parameters. For a modeled workload with $N\geq32$, $dN=N-N_{0}$ is the number of additional sample rows beyond that measured base. Here, $Q$ is the retained $q$-column covariate basis and $Y$ is the phenotype matrix. Residualizing the $K$ phenotype columns against $Q$ requires two matrix products. $Q^{\top}Y$ performs approximately $2dNqK$ FLOPs and $Q\left( Q^{\top}Y \right)$ performs another $2dNqK$, because each output dot product contains $dN$ multiplications and approximately $dN$ additions. Their sum is therefore $4dNqK$ FLOPs.

$$T_{\mathrm{design}}=\frac{t_{tiny,CPU}}{f_{\mathrm{CPU}}}+\frac{t_{tiny,nonCPU}}{f_{\mathrm{GPU}}}+\frac{\max\left( 4dNqK/R_{FP32}, 4dN\left( 6q+18K+4 \right)/R_{\mathrm{HBM}} \right)}{f_{\mathrm{GPU}}}$$

$$+\frac{4dN\left( 2q+3K \right)}{R_{H2D}}+\frac{4dNK}{R_{D2H}}$$

Here $f_{\mathrm{GPU}}$ is the available GPU fraction. The device-memory term $4dN\left( 6q+18K+4 \right)$ is calculated as 4 bytes per float32 value $\times$ $dN$ additional samples $\times$ [six covariate-basis-related value movements for each of $q$ retained directions + eighteen phenotype/intermediate movements for each of $K$ phenotypes + four scalar or auxiliary movements]. The coefficients 6, 18 and 4 aggregate logical reads and writes across centering, projection, standardization, concatenation and sum-of-squares formation. They are source-informed logical traffic rather than asserted physical HBM transactions after caching or kernel fusion. Arithmetic time is $\frac{4dNqK}{R_{FP32}}$ and memory time is $\frac{4dN\left( 6q+18K+4 \right)}{R_{\mathrm{HBM}}}$. The max() operation selects the slower bound because both constrain the same GPU work, and division by $f_{\mathrm{GPU}}$ accounts for partial GPU availability. The host-to-device term $4dN\left( 2q+3K \right)$ is 4 bytes $\times$ $dN$ samples $\times$ two retained-basis-sized plus three phenotype-sized transferred arrays. The device-to-host term $4dNK$ is 4 bytes $\times$ $dN$ samples $\times$ $K$ returned phenotype values. The measured tiny-call CPU and non-CPU services are added separately because they are fixed setup primitives rather than scaled products. $R_{FP32}$ and $R_{\mathrm{HBM}}$ are the independently measured float32 arithmetic and GPU-memory rates. $R_{H2D}$ and $R_{D2H}$ are the independently measured transfer rates. The terms $t_{tiny,CPU}$ and $t_{tiny,nonCPU}$ are the measured CPU and non-CPU times of the small base setup call.

#### **S4.3.3 TorchGWAS finite scan, significance and output**

The TorchGWAS input model accounts for each native format separately. BED transfers packed two-bit rows, or $\mathrm{ceil}\left( N/4 \right)$ bytes for each variant, and charges GPU unpacking and missing-value handling. Hard-call PGEN charges CPU record and linkage-disequilibrium decoding and transfers one-byte integer calls, or $N$ bytes for each variant. Dosage PGEN charges CPU dosage decoding and transfers float32 dosages, or $4N$ bytes for each variant. BGEN charges CPU record parsing and the selected decoder path. The direct GPU path transfers compressed probability records and charges GPU decompression and probability conversion, whereas the CPU fallback transfers $4N$ bytes of float32 dosages for each variant. Here, $N$ is the number of analyzed samples. Actual file and record byte counts are used for storage service, so variable-length PGEN and BGEN inputs are not estimated from a generic compression ratio.

For every input path, the predictor separately counts storage reads, host-memory traffic, host-to-device transfer, CPU and GPU decoder service, common association work, result return, output writing and pipeline fill and drain. Independent stages may overlap only when the finite dependency graph permits it. GPU decoding is added to association service because both use the same device, and the configured worker, queue and buffer limits prevent ideal or unbounded overlap.

The following equations instantiate the hard-call PGEN path used for the crossover comparison. For block $i$, $M$ is the total variant count, $B$ is the chunk capacity and $D$ is the pipeline depth. The actual variant count is $b_{i}=min\left( B,M-iB \right)$, and $f_{i}=\frac{b_{i}}{M}$ is the block’s share of total variant work. Under the stated uniform encoded-work scenario, the block receives $f_{i}$ of the total PGEN decoder work and encoded record payload. Let $n_{\mathrm{decoder}}$ be the configured number of decoder workers. The first $W=min\left( \mathrm{ceil}\left( M/B \right),n_{\mathrm{decoder}},D \right)$ blocks also initialize one decoder-worker index each:

$$U_{decode,i}=f_{i}U_{decode,total}+I\left( i<W \right)Mu_{\mathrm{index}}$$

$$H_{decode,i}=2f_{i}B_{\mathrm{record}}+2\mathrm{ceil}\left( N/4 \right)b_{i}+2Nb_{i}$$

$$T_{decode,i}=max\left( U_{decode,i}/f_{\mathrm{CPU}}, H_{decode,i}/R_{\mathrm{DRAM}} \right)$$

$$T_{read,i}=\frac{f_{i}B_{\mathrm{record}}}{R_{r}}, T_{H2D,i}=\frac{Nb_{i}}{R_{H2D}}$$

The fraction $f_{i}$ = $b_{i}$/$M$ assigns block $i$ its $b_{i}$-variant share of total decoder work and encoded payload. Thus $f_{i}U_{decode,total}$ is that fraction $\times$ the full-input decoder CPU service. For each of the first $W$ worker-initializing blocks, $I\left( i<W \right)=1$ and $M$ $u_{\mathrm{index}}$ is $M$ index records $\times$ CPU-seconds per record for one complete index construction. For later blocks the indicator is zero. In $H_{decode,i}$, $2f_{i}B_{\mathrm{record}}$ counts one write and one read of the block’s encoded-record staging bytes. Because four two-bit hard calls fit in one byte, $\mathrm{ceil}\left( N/4 \right)b_{i}$ is the packed size for $b_{i}$ variants, and the leading 2 again counts its write and subsequent read. The decoded matrix contains $Nb_{i}$ one-byte int8 calls, so producing and then consuming it contributes $2Nb_{i}$ logical bytes. $T_{decode,i}$ takes the larger of CPU service $\frac{U_{decode,i}}{f_{\mathrm{CPU}}}$ and memory service $\frac{H_{decode,i}}{R_{\mathrm{DRAM}}}$ because they limit the same decoding stage. $T_{read,i}$ divides the block’s $f_{i}$ share of encoded file bytes by input rate $R_{r}$. $T_{H2D,i}$ divides $Nb_{i}$ transferred bytes ($N$ samples $\times$ $b_{i}$ variants $\times$ one byte) by host-to-device bandwidth $R_{H2D}$. Here, $U_{decode,total}$ is the decoder CPU service for the full input, $u_{\mathrm{index}}$ is the measured CPU service for one index record and $B_{\mathrm{record}}$ is the encoded record payload. $R_{\mathrm{DRAM}}$, $R_{r}$ and $R_{H2D}$ are the independently measured host-memory, input and host-to-device rates. The factor $f_{\mathrm{CPU}}$ is the available fraction of one logical CPU for serial decoder work.

For one block, each of $b_{i}$ variants is paired with $K$ phenotype columns, $q$ retained covariate-basis columns and one intercept column. The association output width is therefore

$$P=K+q+1$$

This gives $b_{i}P$ output dot products. Each dot product reduces across $N$ samples and is counted as $N$ multiplications plus approximately $N$ additions, or $2N$ FLOPs. Multiplying $2N$ FLOPs per output by $b_{i}P$ outputs gives $2Nb_{i}P$ useful FLOPs. Kernel service is based on the larger issued tiled work rather than only this useful count. The split-reduction count $s_{K}$, compiled reduction tile $t_{K}$, variant tile $t_{M}$, output-width tile $t_{P}$ and launch-grid extents $g_{M}$ and $g_{P}$ are taken from the compiled kernel configuration for the modeled shape:

$$N_{\mathrm{issued}}=ceil\left[ N/\left( s_{K}t_{K} \right) \right]s_{K}t_{K}, M_{\mathrm{pad}}=g_{M}t_{M}, P_{\mathrm{pad}}=g_{P}t_{P}$$

$$F_{\mathrm{issued}}=2N_{\mathrm{issued}}M_{\mathrm{pad}}P_{\mathrm{pad}}$$

$N_{\mathrm{issued}}$ rounds the useful sample dimension $N$ upward to a multiple of $s_{K}t_{K}$: $\mathrm{ceil}\left( N/\left( s_{K}t_{K} \right) \right)$ counts required reduction-tile groups and multiplication by $s_{K}t_{K}$ restores the padded number of issued reduction positions. $M_{\mathrm{pad}}=g_{M}t_{M}$ is grid blocks along the variant dimension $\times$ variants issued per tile, and $P_{\mathrm{pad}}=g_{P}t_{P}$ is grid blocks along the output-width dimension $\times$ output columns issued per tile. The product $2N_{\mathrm{issued}}M_{\mathrm{pad}}P_{\mathrm{pad}}$ is two FLOPs per multiply-add position $\times$ padded reduction positions $\times$ padded variant positions $\times$ padded output positions. Here the subscript $K$ in $s_{K}$ and $t_{K}$ follows GEMM reduction-dimension naming and is unrelated to the manuscript phenotype count $K$. Split-reduction workspace reads, writes and additions, grid occupancy, kernel launches, host dispatch and source-derived L2/HBM traffic are also included. For kernel $j$, active multiprocessor fraction is min(1, grid blocks/SM count). Arithmetic and memory limits are combined by a maximum, launch service is added, and stream order starts each kernel no earlier than both its host dispatch and the preceding GPU completion.

Current dense output additionally evaluates one exact two-sided Student-$t$ tail for each of the $b_{i}K$ association cells. Here, $t$ is the Student statistic and $d$ is its residual degrees of freedom. The tensor path converts $t$ and $d$ to float64, forms $x=\frac{d}{\left( d+t^{2} \right)}$, evaluates three lgamma terms and logarithmic front factors, and evaluates the regularized incomplete beta using a fixed 40-iteration Lentz continued fraction. Cells in the reflected branch require the second continued-fraction evaluation before selection. Thus

$$L_{\log P,i}=b_{i}K, T_{GPU,i}=T_{OLS graph,i}+T_{\log P,i}$$

The earlier no-output crossover predictor did not require this dense significance graph. Here, $L_{\log P,i}$ is the number of tail evaluations in block $i$, $T_{\log P,i}$ is their predicted time, $T_{OLS graph,i}$ is the GPU time for the OLS graph and $T_{GPU,i}$ is the sum of OLS and tail-evaluation time. $T_{\log P,i}$ is priced from the source-counted float64 operations, the fixed continued-fraction work, launch service and modeled device-memory traffic using the same device-rate and roofline rules as the OLS graph. It is not assigned zero or hidden in an empirical end-to-end correction.

With dense $-\log_{10}P$, block $i$ returns $4b_{i}K$ bytes each of beta and $t$, $8b_{i}K$ bytes of float64 $-\log_{10}P$, $b_{i}$ status bytes and $4b_{i}$ residual-df bytes. In the next equation, $B_{D2H,i}$ is the total number of bytes returned by block $i$, $T_{D2H,i}$ is the transfer time and $R_{D2H}$ is the independently measured device-to-host rate:

$$B_{D2H,i}=\left( 16K+5 \right)b_{i}, T_{D2H,i}=\frac{B_{D2H,i}}{R_{D2H}}$$

Without $-\log_{10}P$, $B_{D2H,i}=\left( 8K+5 \right)b_{i}$. The current default durable store casts $-\log_{10}P$ to float32 and writes beta.f32, tstat.f32 and neglog10p.f32, for $12MK$ payload bytes. Screening-only output omits beta and writes $8MK$ bytes. The historical full-genome timing experiment wrote beta and $t$ only, totaling $8MK=36.7 \mathrm{GB}$. Its measured write volume is therefore not retroactively replaced by the current three-array contract.

$$A_{\mathrm{out}}=2+I_{\beta}, B_{\mathrm{durable}}=4A_{\mathrm{out}}MK$$

$$B_{\mathrm{block}}=min\left( 16 MiB,max\left[ 1 MiB,4K\min\left( M,B \right) \right] \right)$$

$A_{\mathrm{out}}$ = 2 + $I_{\beta}$ because tstat.f32 and neglog10p.f32 are always stored and beta.f32 adds one array when $I_{\beta}=1$. The durable payload $4A_{\mathrm{out}}MK$ is 4 bytes per float32 value $\times$ $A_{\mathrm{out}}$ arrays $\times$ $M$ variants $\times$ $K$ phenotypes. For one array, the first-chunk payload is $4K\min\left( M,B \right)$: four bytes $\times$ $K$ phenotypes $\times$ the smaller of total variants $M$ and chunk capacity $B$. $B_{\mathrm{block}}$ clamps this value to the interval from 1 to 16 MiB. Let $Q_{w}$ be the writer queue depth. Each array owns $Q_{w}$ queued blocks plus one block currently being filled, so $A_{\mathrm{out}}\left( Q_{w}+1 \right)B_{\mathrm{block}}$ is arrays $\times$ allocated blocks per array $\times$ bytes per block. A staged byte is read once from the incoming result and written once into a staging block, contributing two logical host-memory bytes. A borrowed complete block avoids this copy. For a queued block, $\frac{B_{\mathrm{copy}}u_{\mathrm{copy}}}{f_{\mathrm{CPU}}}$ is copied bytes $\times$ CPU-seconds per byte divided by the serial CPU share, whereas $\frac{B_{\mathrm{queued}}}{R_{w}}$ is written bytes divided by sustained output bandwidth. Copy and write are represented as separate finite-graph nodes rather than collapsed into a single $T_{\mathrm{write}}$ term. Here, $B_{\mathrm{copy}}$ is the number of copied staging bytes, $B_{\mathrm{queued}}$ is the number of queued output bytes, $u_{\mathrm{copy}}$ is the measured CPU service for one copied byte, $f_{\mathrm{CPU}}$ is the available fraction of one logical CPU and $R_{w}$ is the independently measured output rate.

The scan graph contains decoder submission, storage read, decode, publication, host-to-device submission and transfer, ordered host API dispatch and GPU kernels, result submission and device-to-host transfer, host finishing, writer copy, writer write and close. A node starts only after all predecessors complete and a buffer slot is available. The finite graph uses proportional active-demand fluid sharing for CPU, host DRAM, input and output capacities. Let A be the set of active nodes. For node $j$ and resource $r$, $d_{jr}$ is the nominal demand rate in resource units per nominal second, $C_{r}$ is the available capacity in the same resource units per second, and $s_{j}$ is the remaining nominal service time in seconds. The total active demand is

$$D_{r}=\sum_{j\in A} d_{jr}$$

$$\lambda_{j}=min\left( 1,\min_{r: d_{jr}>0}C_{r}/D_{r} \right)$$

$$\Delta=\frac{\min_{j}s_{j}}{\lambda_{j}}, s_{j}\leftarrow s_{j}-\lambda_{j}\Delta$$

Thus all nodes sharing an oversubscribed resource slow by $\frac{C_{r}}{D_{r}}$, and a node using multiple resources is governed by its smallest fraction. The scheduler advances to the next completion, releases completed demands, activates newly eligible nodes and recomputes the fractions. This is proportional fluid sharing, not an operating-system scheduling simulation. GPU availability is incorporated into the device-service times, and GPU execution order is represented by graph dependencies rather than by this shared-resource summation. The scan-and-close time is the final graph completion time, not the sum of independently reported stage clocks.

#### **S4.3.4 PLINK 2.0 predictor**

The PLINK 2.0 candidate modeled the hard-call PGEN --glm branch with one analyzed phenotype ($K=1$), 48 requested threads and 47 calculation workers in the H100 profile. Its total predicted runtime is

$$T_{\mathrm{PLINK}}=T_{\mathrm{setup}}+T_{\mathrm{scan}}$$

No process-launch term is included. Cold setup includes measured metadata bytes divided by the input rate $R_{r}$, token scanning, selected numeric conversion, retained-field memory traffic, ordered-ID comparison work and fixed-design arithmetic:

$$U_{\mathrm{parse}}=\frac{L_{\mathrm{token}}u_{token16}}{16}+N\left( c+1 \right)u_{\mathrm{double}}+Mu_{\mathrm{uint}}$$

$$U_{\mathrm{match}}=\frac{L_{\mathrm{match}}u_{token16}}{16}, F_{\mathrm{setup}}=2N\left( c+1 \right)\left( c+2 \right)+2N\left( c+1 \right)+2\left( c+1 \right)^{3}$$

Here $K_{\mathrm{file}}$ is the number of phenotype-value columns parsed from the phenotype input file, excluding identifier columns, whereas $K$ is the number of phenotypes analyzed in the association scan. In the current synthetic crossover profile $K_{\mathrm{file}}=32$ and $K=1$. For retained metadata traffic, $F_{\mathrm{table}}$ is the total number of delimited fields across the PVAR, PSAM, phenotype and covariate tables. The model assigns an approximate 64 logical bytes of retained-object and metadata traffic per parsed field. This is traffic rather than resident memory usage. For the current synthetic benchmark profile,

$$F_{\mathrm{table}}=5M+3N+N\left( K_{\mathrm{file}}+2 \right)+N\left( c+2 \right)$$

$$T_{metadata,memory}=\frac{64F_{\mathrm{table}}}{R_{\mathrm{DRAM}}}$$

$$T_{\mathrm{setup}}=\frac{B_{\mathrm{metadata}}}{R_{r}}+\frac{\left[ U_{\mathrm{parse}}+U_{\mathrm{match}}+F_{\mathrm{setup}}/R_{FP64}+T_{metadata,memory} \right]}{f_{\mathrm{CPU}}}$$

With $K_{\mathrm{file}}=32$ and $c=8$, $F_{\mathrm{table}}=5M+47N$. The implementation places $T_{metadata,memory}$ inside the same serial setup bracket as the parser, matching and arithmetic services, so the entire bracket is divided by $f_{\mathrm{CPU}}$.

In the PLINK 2.0 parser model, a token is one delimited metadata field or identifier consumed by the text parser, not a word or a model token. $L_{\mathrm{token}}$ counts the characters in the parsed fields plus one delimiter allowance for each field. Dividing $L_{\mathrm{token}}$ by 16 converts that count into 16-character units. The independently measured $u_{token16}$ primitive is the CPU service for one such unit, so $\left( L_{\mathrm{token}}/16 \right)u_{token16}$ is the token-scanning CPU service. $L_{\mathrm{match}}$ is the corresponding character count for identifier matching. $N\left( c+1 \right)$ is $N$ sample rows multiplied by $c$ covariates plus one phenotype value per row, and multiplication by $u_{\mathrm{double}}$ prices those numeric conversions. $M$ $u_{\mathrm{uint}}$ is one unsigned-integer conversion per variant. In $F_{\mathrm{setup}}$, $c+1$ is the intercept-plus-covariate width and $c+2$ additionally includes the tested genotype. The term $2N\left( c+1 \right)\left( c+2 \right)$ counts multiply-add work for cross-products between these columns over $N$ samples, $2N\left( c+1 \right)$ counts the remaining sample-by-design transformations and $2\left( c+1 \right)^{3}$ counts the small dense factorization and update. Each FLOP count is divided by $R_{FP64}$. Parsing, retained-memory, matching and arithmetic services occur sequentially during setup. $R_{FP64}$ is the independently measured float64 arithmetic rate and $f_{\mathrm{CPU}}$ is the available fraction of one logical CPU for serial work.

For the synthetic workload model, $\rho$ is the specified iid missing-call probability rather than an empirical coefficient fitted from a real genotype input. The crossover profile uses $\rho=0.001$, or 0.1%, and the generated crossover files have realized missing fractions close to 0.001. The complete-variant probability is

$$p_{\mathrm{complete}}=\left( 1-\rho\right)^{N}$$

This is the probability that all $N$ samples have observed calls for a variant. For a real dataset, the empirical overall missing fraction can be supplied as an approximation, but exact PLINK branch counts would also depend on the per-variant completeness and transition pattern. A restart segment is a contiguous variant block assigned to one calculation worker, and $S_{\mathrm{restart}}$ is the number of worker or block restart segments. The expected branch counts are

$$M_{\mathrm{gram}}=M\left( 1-p_{\mathrm{complete}} \right)$$

$$M_{\mathrm{opener}}=S_{\mathrm{restart}}p_{\mathrm{complete}}+\left( M-S_{\mathrm{restart}} \right)p_{\mathrm{complete}}\left( 1-p_{\mathrm{complete}} \right)$$

$$M_{\mathrm{sparse}}=\left( M-S_{\mathrm{restart}} \right)p_{\mathrm{complete}}^{2}, N_{\mathrm{observed}}=\frac{N\left( M_{\mathrm{gram}}-M\rho\right)}{M_{\mathrm{gram}}}$$

The complete-variant probability $p_{\mathrm{complete}}=\left( 1-\rho\right)^{N}$ is the probability that all $N$ independently modeled calls are observed. Therefore $M\left( 1-p_{\mathrm{complete}} \right)$ is the expected number $M_{\mathrm{gram}}$ of incomplete variants. Among complete variants, $S_{\mathrm{restart}}p_{\mathrm{complete}}$ counts complete variants at worker/block restart positions, and $\left( M-S_{\mathrm{restart}} \right)p_{\mathrm{complete}}\left( 1-p_{\mathrm{complete}} \right)$ counts complete non-restart variants whose predecessor is incomplete. Together these form $M_{\mathrm{opener}}$. The product $\left( M-S_{\mathrm{restart}} \right)p_{\mathrm{complete}}^{2}$ counts non-restart variants for which both the current and preceding variants are complete, giving $M_{\mathrm{sparse}}$. For the Gram path, $N\left( M_{\mathrm{gram}}-M\rho\right)$ subtracts the expected missing calls $NM\rho$ from the $NM_{\mathrm{gram}}$ call positions assigned to incomplete variants. Division by $M_{\mathrm{gram}}$ gives the conditional mean $N_{\mathrm{observed}}$ per Gram variant.

The Gram branch constructs a nonmissing mask, expands packed calls, gathers the observed design and phenotype, forms the Gram matrix and genotype-phenotype products, solves the small system and evaluates post-regression quantities. Complete-variant opener and sparse-update branches use rank-one update paths. For each source stage, instruction service, arithmetic FLOPs/rate and ideal-LRU L1/L2/L3/DRAM traffic/rate were maximized. Stages within a branch were then summed. The first variant on each restarted worker retained its first-touch-minus-steady increment.

$$U_{j}=\frac{\left[ \sum_{b} M_{b}T_{b,j} \right]}{n_{\mathrm{workers}}}+\frac{U_{\mathrm{decode}}}{n_{\mathrm{workers}}}$$

$$+\frac{S_{\mathrm{restart}}\max\left( 0,T_{gram,first,j}-T_{gram,steady,j} \right)}{n_{\mathrm{workers}}}$$

$$T_{compute pool}=max\left( \max_{j}U_{j}/f_{\mathrm{CPU}}, \left( \sum_{j} U_{j} \right)/C_{\mathrm{CPU}} \right)$$

For worker $j$, $U_{j}$ is the modeled CPU service assigned to that worker. Each product $M_{b}T_{b,j}$ is the expected number $M_{b}$ of variants taking branch b multiplied by worker $j$’s modeled per-variant service $T_{b,j}$. $n_{\mathrm{workers}}$ is the number of calculation workers, $U_{\mathrm{decode}}$ is total decoder service and $S_{\mathrm{restart}}$ is the number of worker or block restarts. Summing over branches and dividing by $n_{\mathrm{workers}}$ distributes nominal work across the calculation threads. The restart term is the restart count multiplied by the positive first-touch excess per restart and divided by the worker count. The pool must satisfy both the busiest-worker time $\frac{\max_{j}\left( U_{j} \right)}{f_{\mathrm{CPU}}}$ and the aggregate-capacity time $\frac{\left( \sum_{j} U_{j} \right)}{C_{\mathrm{CPU}}}$, so their maximum is used. Here $f_{\mathrm{CPU}}$ is the available fraction of one logical CPU and $C_{\mathrm{CPU}}$ is the process-wide CPU capacity in core-equivalents.

PGEN decoder primitive counts were priced independently and maximized against the modeled L2 traffic of encoded input, packed output, copy, fill and inversion paths. Per-variant formatting contained four floating-point conversions, one line-format call, one $t$-tail call and two unsigned-integer conversions. Output characters were counted from identifier lengths, numeric fields, sample-count width and delimiters. Block-level read, compute and format/write services were scheduled in PLINK's read-current, join-previous, launch-current and format-previous order. The final scan was the maximum of the finite schedule, total CPU work/$C_{\mathrm{CPU}}$ and total modeled DRAM bytes/$R_{\mathrm{DRAM}}$. In the final resource bounds, $C_{\mathrm{CPU}}$ is the process-wide CPU capacity and $R_{\mathrm{DRAM}}$ is the independently measured host-memory bandwidth.

#### **S4.3.5 fastGWA-lr predictor**

The fastGWA-lr candidate modeled the tested GCTA v1.95.3 hard-call path with one analyzed phenotype ($K=1$), one sequential reader and one effectively serial analysis consumer. Its total predicted runtime is

$$T_{\mathrm{fastGWA}}=T_{\mathrm{setup}}+T_{\mathrm{scan}}$$

No process-launch term is included. Cold setup includes measured metadata bytes divided by the input rate $R_{r}$ and parser, retained-string, numeric-conversion, sample-matching, arithmetic and memory terms. Here, $c$ is the number of non-intercept covariates and the fixed-design width including the intercept is

$$p_{X}=c+1$$

The per-row and per-field primitives $u_{\mathrm{split}\left( t \right)}$, $u_{\mathrm{short}}$, $u_{\mathrm{construct}}$, $u_{\mathrm{compare}}$, $u_{\mathrm{strtod}}$ and $u_{\mathrm{stoi}}$ are independently measured CPU services. $S_{\mathrm{string}}$ and $S_{\mathrm{integer}}$ are comparison counts taken from the sample-matching control-flow trace used to build the predictor. $K_{\mathrm{file}}$ retains the definition above.

$$U_{\mathrm{text}}=\sum_{t} \mathrm{rows}_{t}u_{\mathrm{split}\left( t \right)}$$

$$U_{\mathrm{copy}}=\sum_{t} \left( \mathrm{short}_{t}u_{\mathrm{short}}+\mathrm{long}_{t}u_{\mathrm{construct}} \right)+3Mu_{\mathrm{short}}$$

$$U_{\mathrm{numeric}}=N\left( K_{\mathrm{file}}+c \right)u_{\mathrm{strtod}}+2Mu_{\mathrm{stoi}}$$

$$U_{\mathrm{matching}}=2S_{\mathrm{string}}u_{\mathrm{compare}}+3S_{\mathrm{integer}}u_{\mathrm{compare}}+\left[ 2\left( N-1 \right)+3N \right]u_{\mathrm{compare}}$$

$$F_{\mathrm{setup}}=4Np_{X}^{2}+\left( 2/3 \right)p_{X}^{3}+4Np_{X}, H_{\mathrm{setup}}=64F_{\mathrm{table}}+16Np_{X}$$

$$T_{\mathrm{setup}}=\frac{B_{\mathrm{metadata}}}{R_{r}}+\left[ U_{\mathrm{text}}+U_{\mathrm{copy}}+U_{\mathrm{numeric}}+U_{\mathrm{matching}} \right.$$

$$\frac{\left. +max\left( F_{\mathrm{setup}}/R_{FP64},H_{\mathrm{setup}}/R_{\mathrm{DRAM}} \right) \right]}{f_{\mathrm{CPU}}}$$

For fastGWA-lr, every setup product is again a count $\times$ unit service. In $U_{\mathrm{text}}$, $\mathrm{rows}_{t}$ is the number of rows in input table $t$ and $u_{\mathrm{split}\left( t \right)}$ is CPU-seconds per parsed row. Summation covers all tables. In $U_{\mathrm{copy}}$, $\mathrm{short}_{t}$ and $\mathrm{long}_{t}$ are retained short and long fields multiplied by their respective per-field copy or construction services, and $3M$ $u_{\mathrm{short}}$ counts three retained marker strings per variant. In $U_{\mathrm{numeric}}$, $N\left( K_{\mathrm{file}}+c \right)$ is $N$ sample rows $\times$ all phenotype-file and covariate numeric columns, while $2M$ is two integer fields per variant. In $U_{\mathrm{matching}}$, $S_{\mathrm{string}}$ and $S_{\mathrm{integer}}$ are the saved counts of string and integer comparisons in the sample-matching trace. The bracket $2\left( N-1 \right)+3N$ counts two adjacent-row passes plus three complete sample passes. With $p_{X}=c+1$, $4Np_{X}^{2}$ counts the leading sample-by-design dense work, $\left( 2/3 \right)p_{X}^{3}$ the small factorization and $4Np_{X}$ the remaining transformations. $F_{\mathrm{table}}$ is the number of retained fields. $H_{\mathrm{setup}}=64F_{\mathrm{table}}+16Np_{X}$ is 64 logical bytes per retained field plus two eight-byte movements for each of $Np_{X}$ double-precision design entries. The larger of $\frac{F_{\mathrm{setup}}}{R_{FP64}}$ and $\frac{H_{\mathrm{setup}}}{R_{\mathrm{DRAM}}}$ prices the same dense setup work. The other parser and matching services are added, and division by $f_{\mathrm{CPU}}$ converts serial CPU service to elapsed time. Here, $R_{FP64}$ is the independently measured float64 arithmetic rate, $R_{\mathrm{DRAM}}$ is the independently measured host-memory bandwidth and $f_{\mathrm{CPU}}$ is the available fraction of one logical CPU for serial work.

The decoder summed record-dispatch, variable-length integer, one-bit, group-header, category-extraction and inversion primitives and maximized that instruction service against packed-input and copy traffic divided by L2 bandwidth. In the reader expression, $B_{\mathrm{PGEN}}$ is the PGEN file size, $R_{r}$ is the measured input rate, $U_{\mathrm{decode}}$ is decoder CPU service and $f_{\mathrm{CPU}}$ is the available fraction of one logical CPU for serial work. The per-variant analysis trace contained packed-genotype counting, genotype allocation and zeroing, expansion, the Eigen alias-safe temporary copy, phenotype residualization, result copying and the genotype and genotype-phenotype inner products. Each stage used the maximum of its instruction, arithmetic and cache-memory limits. The ten stages were summed. The ideal-LRU trace was iterated to a stationary cost, giving

$$U_{\mathrm{math}}=T_{variant,first}+\left( M-1 \right)T_{variant,steady}, U_{\mathrm{tail}}=Mu_{\chi^{2}\text{-tail}}$$

$$U_{\mathrm{format}}=M\left[ 4u_{ostream,double}+u_{ostream,uint}+2u_{toString,uint}+8u_{short,string} \right]$$

In the fastGWA-lr analysis products, $T_{variant,first}$ is paid once and $\left( M-1 \right)T_{variant,steady}$ is the number of remaining variants $\times$ steady per-variant service. $U_{\mathrm{tail}}=Mu_{\chi^{2}\text{-tail}}$ is $M$ variants $\times$ one measured chi-square-tail call per variant. The bracket in $U_{\mathrm{format}}$ is per-variant formatting service: four floating-point stream insertions, one unsigned-integer insertion, two integer-to-string conversions and eight short-string operations. Multiplication by $M$ gives total formatting CPU-seconds. Decoder, analysis, tail and formatting services enter the finite reader/consumer schedule and the whole-process CPU floor. They are not separate complete-runtime fitting coefficients.

Variants were divided into 1,024-variant blocks and passed through a three-slot queue. For block $i$, $r_{start,i}$ and $r_{end,i}$ are the reader start and end times, $a_{start,i}$ and $a_{end,i}$ are the analysis start and end times, and $r_{service,i}$ and $a_{service,i}$ are the corresponding service times:

$$r_{start,i}=max\left( r_{end,i-1},a_{end,i-3} \right), r_{end,i}=r_{start,i}+r_{service,i}$$

$$a_{start,i}=max\left( a_{end,i-1},r_{end,i} \right), a_{end,i}=a_{start,i}+a_{service,i}$$

The first absent predecessor was treated as time zero, and the final $a_{end,i}$ was the finite-queue makespan. In the resource floor, $U_{\mathrm{decode}}$, $U_{\mathrm{math}}$, $U_{\mathrm{format}}$ and $U_{\mathrm{tail}}$ are total decoder, association, formatting and tail-evaluation CPU services. $C_{\mathrm{CPU}}$ is the process-wide CPU capacity, $H_{\mathrm{DRAM}}$ is total modeled host-memory traffic and $R_{\mathrm{DRAM}}$ is the independently measured host-memory bandwidth. The reported scan was the maximum of the finite-queue makespan, total CPU service divided by $C_{\mathrm{CPU}}$ and $H_{\mathrm{DRAM}}$/$R_{\mathrm{DRAM}}$. This preserves reader-analysis overlap while preventing the effectively serial association loop from receiving ideal multithreaded scaling.

#### **S4.3.6 Hardware profiles, crossover search and interpretation**

Storage, host-memory, transfer, CPU primitive, GPU arithmetic, GPU-memory and launch/dispatch rates were measured independently of complete association runtimes on each hardware platform. Primitive CPU rates were medians of repeated calls to the corresponding parser, quality-control, basis, index, decoder, conversion, formatting or allocation routine. For the Figure 2 H100 scenario, $f_{\mathrm{CPU}}=f_{\mathrm{GPU}}=1$ represent unshared quiet-host execution. The GPU was verified idle before the run, and its measured component rates reflect effective rather than specification-sheet performance. PLINK uses $C_{\mathrm{CPU}}=40.125$ core-equivalents, the median of 41.75 and 38.50 observed in two separate quiet-host $K=2$ PLINK runs, with core-equivalents calculated as process CPU time divided by wall time. TorchGWAS and fastGWA retain 48 process-wide core-equivalents, although the tested fastGWA association loop is effectively serial. These quantities are profile inputs, not coefficients fitted to the crossover measurements. Complete runs were reserved for validation and measured bracketing near predicted transitions.

For a fixed sample count $N$ and comparator $h$, the discrete search evaluated the predicted runtime difference

$$\Delta_{h}\left( M \right)=T_{\mathrm{Torch}}\left( N,M \right)-T_{h}\left( N,M \right)$$

where $M$ is the variant count, the first term is the predicted TorchGWAS runtime and the second is the predicted comparator runtime. Starting at one TorchGWAS chunk, $M$ was doubled until the difference changed from positive to non-positive and was then bisected on the chunk grid. If the difference was positive at the lower endpoint $M_{\mathrm{low}}$ and non-positive at the upper endpoint $M_{\mathrm{high}}$, the plotted boundary was the logarithmic midpoint

$$M_{\mathrm{curve}}=\sqrt{M_{\mathrm{low}}M_{\mathrm{high}}}$$

$M_{\mathrm{curve}}$ is the plotted crossover variant count. The product $M_{\mathrm{low}}M_{\mathrm{high}}$ multiplies the lower and upper variant-count endpoints of the final crossover bracket, and its square root is their geometric mean. This is the midpoint on the logarithmic $M$ axis used in the figure, not an additional performance model or a confidence interval. Each prediction also searched larger $M$ for a later reversal. The H100 PLINK 2.0 profile used 47 calculation workers and a shared 60-MiB reference-stream last-level-cache scenario. The alternative equal-partition cache scenario was retained as a sensitivity analysis. The tested fastGWA-lr profile retained one analysis consumer. Because $N$, $M$, $K$, input encoding, missingness, output fields, cache state and hardware capacities do not scale identically, neither $MN$ nor $KMN$ is a universal crossover constant.

### S4.4 Scope and limitations

These benchmarks cover quantitative-trait fixed-effect OLS on a single CUDA-capable GPU. TorchGWAS 1.0 does not fit a genomic random effect, so studies retaining related individuals require an appropriate mixed-model method. The phenotype panel must fit device memory for a scan, and larger panels must be split into groups. Multi-GPU scaling is not evaluated here. The reported statistical concordance experiments use complete genotypes, so the missing-value bookkeeping in S1.2 is not independently validated by those experiments. The reported −log10P concordance establishes accuracy over the tested range, but extreme-tail values beyond that range were not separately benchmarked.

# Supplementary Results

## S5. Statistical agreement, calibration and power

TorchGWAS float64 reproduced direct full OLS to numerical precision across all 192 tests. Maximum absolute differences were 2.22 × 10⁻¹⁵ for β, 6.66 × 10⁻¹⁶ for SE, 1.14 × 10⁻¹³ for t and 5.66 × 10⁻¹⁵ for P. Float32 retained 100% effect-direction and genome-wide-significance agreement. Its maximum absolute t and −log10P differences were 9.24 × 10⁻⁵ and 1.17 × 10⁻⁴. Across 500,036 allele-harmonized tests, TorchGWAS and PLINK 2.0 t statistics had Pearson 1 − r = 1.34 × 10⁻¹², where r is the Pearson correlation coefficient, with a median absolute difference of 3.31 × 10⁻⁷ and a maximum absolute difference of 4.89 × 10⁻⁵ (Supplementary Fig. S1 and Table S2).

Across the structured-null settings, TorchGWAS and PLINK 2.0 produced identical false-positive counts at every evaluated threshold. Here, α denotes the nominal rejection threshold. At α = 0.05, TorchGWAS rates ranged from 0.049325 to 0.049993. At α = 10⁻³, rates ranged from 0.000936 to 0.001035. The no-PC model became increasingly inflated as population structure explained more phenotype variance, whereas the phenotype-only formula was mildly conservative (Supplementary Fig. S2 and Table S3).

TorchGWAS, direct full OLS and PLINK 2.0 produced identical detection counts in all 480 power conditions, with aggregate mean power 0.8758 and median power 1.000. Condition-level estimates remain discrete because each condition used 20 replicates (Supplementary Fig. S3 and Table S4).

## S6. Pipeline performance and crossover evidence

With 512 phenotypes (K = 512), the full-genome cold-cache experiment completed 4.57 billion associations in median end-to-end times of 28.46 s for BED, 29.13 s for hard-call PGEN, 51.48 s for BGEN and 58.95 s for dosage PGEN, including the 36.7-GB output. BED and hard-call PGEN were indistinguishable within their two-run spreads. BGEN was dominated partly by cold metadata opening, whereas dosage PGEN required substantially larger host and device staging buffers (Supplementary Table S5).

TorchGWAS was 1.31–1.56-fold faster than tensorQTL at every evaluated K in the matched no-output experiment. For the single-phenotype CPU comparisons, the transition varied with N rather than following a fixed MN threshold. Across all five H100 profiles in Supplementary Table S6, the predicted crossovers fell inside their measured brackets. These are measured transition intervals, not confidence intervals. No single MN or KMN threshold is universal (Supplementary Table S6 and Fig. 2B and C).

# Supplementary Tables

## Supplementary Table S1. Statistical and implementation definitions

| **Quantity** | **Definition** | **Implementation meaning** |
| --- | --- | --- |
| Covariate basis | Q from rank-revealing SVD. Numerical rank q | Redundant covariates do not consume degrees of freedom |
| Phenotype | Centered, projected off Q and scaled by the post-projection SD across analyzed samples (denominator N) | One processed phenotype matrix is shared across variant blocks |
| Genotype | Centered dosage in the declared effect-allele direction | Genotype is not divided by its SD. β is per dosage copy |
| Residual genotype SS | $g^{\top}g-\parallel Q^{\top}g\parallel^{2}$ | Exact covariate-adjusted denominator, where g is the centered genotype vector and Q is the orthonormal covariate basis. Residualized g is not materialized |
| Coefficient | $\hat{\beta}=\frac{g^{\top}y^{\text{*}}}{g^{\top}g-\parallel Q^{\top}g\parallel^{2}}$ | Estimated genotype effect. The denominator is the residual genotype sum of squares defined above. |
| Standard error | $\mathrm{SE}\left( \hat{\beta} \right)=\sqrt{\frac{RSS/d}{g^{\top}g-\parallel Q^{\top}g\parallel^{2}}}$ | RSS is the regression residual sum of squares, d is the residual degrees of freedom and residual genotype SS is the covariate-adjusted denominator |
| Test statistic | $t=\frac{\hat{\beta}}{\mathrm{SE}\left( \hat{\beta} \right)}$ | The coefficient standard error is used in the denominator. The sign follows the declared effect allele. |
| Missing values | Observed-only mean. Zero contribution after centering | N is the number of aligned samples, q is the numerical covariate rank, and n_g and n_y are observed genotype and phenotype counts. d_g = n_g−q−2, d_y = n_y−q−2 and d_0 = N−q−2. q excludes the intercept, so the subtraction of 2 accounts for the intercept and tested genotype. |
| Reference-path effective df | $d_{\mathrm{eff}}=\frac{d_{g}d_{y}}{d_{0}}$ | N is the number of aligned samples, q is the numerical covariate rank, and n_g and n_y are observed genotype and phenotype counts. d_g = n_g−q−2, d_y = n_y−q−2 and d_0 = N−q−2 |
| Two-sided tail | $I_{x}\left( d/2,1/2 \right), x=\frac{d}{\left( d+t^{2} \right)}$ | t is the observed Student statistic, d is its residual degrees of freedom and I_x is the regularized incomplete beta function. −log10P is evaluated in log space |
| Dense output | float32 β, t and −log10P arrays | The default store uses 4 bytes for each of β, t and −log10P, giving 12 bytes per association. Screening-only output omits β and uses 8 bytes per association. No separate P-value matrix is stored. |

N, M and K denote sample, variant and phenotype counts, respectively. B denotes variants in one streamed block.

## Supplementary Table S2. Numerical concordance

| **Validation** | **Tests** | **Comparison** | **Result** |
| --- | --- | --- | --- |
| Direct OLS | 192 | TorchGWAS float64 | Maximum absolute difference \|Δ\| (TorchGWAS minus direct OLS): β 2.22e−15. SE 6.66e−16. t 1.14e−13. P 5.66e−15 |
| Direct OLS | 192 | TorchGWAS float32 | Maximum absolute TorchGWAS-minus-direct-OLS difference: \|Δt\| 9.24e−5 and \|Δ(−log10P)\| 1.17e−4. |
| PLINK 2.0 audit | 500,036 | Allele-aligned t statistics | Pearson 1−r 1.34e−12, where r is the Pearson correlation coefficient. Median \|Δt\| 3.31e−7 and maximum \|Δt\| 4.89e−5, where Δt is TorchGWAS minus PLINK 2.0 |

GWS denotes genome-wide significance at P < 5 × 10⁻⁸. Direct-OLS values are absolute differences on the aligned TorchGWAS scale.

## Supplementary Table S3. Structured-population null calibration

| **PC variance** | **Method** | **Rate at α = 0.05** | **Rate at α = 10⁻³** | **Median λGC (genomic inflation factor)** |
| --- | --- | --- | --- | --- |
| 0% | TorchGWAS | 0.049993 | 0.000937 | 0.998 |
| 0% | PLINK 2.0 OLS | 0.049993 | 0.000937 | 0.998 |
| 0% | Phenotype-only | 0.042284 | 0.000773 | 0.846 |
| 0% | No-PC | 0.050164 | 0.001011 | 0.997 |
| 5% | TorchGWAS | 0.049857 | 0.000936 | 0.992 |
| 5% | PLINK 2.0 OLS | 0.049857 | 0.000936 | 0.992 |
| 5% | Phenotype-only | 0.042210 | 0.000731 | 0.840 |
| 5% | No-PC | 0.129028 | 0.025245 | 1.464 |
| 20% | TorchGWAS | 0.049956 | 0.001035 | 1.007 |
| 20% | PLINK 2.0 OLS | 0.049956 | 0.001035 | 1.007 |
| 20% | Phenotype-only | 0.042250 | 0.000831 | 0.855 |
| 20% | No-PC | 0.207391 | 0.087668 | 2.012 |
| 40% | TorchGWAS | 0.049325 | 0.000946 | 0.999 |
| 40% | PLINK 2.0 OLS | 0.049325 | 0.000946 | 0.999 |
| 40% | Phenotype-only | 0.041480 | 0.000799 | 0.849 |
| 40% | No-PC | 0.296198 | 0.163243 | 2.895 |

Each row contains 800,000 null tests. Tests within a setting share genotypes and variants, so rates are reported descriptively without binomial confidence intervals. Here, α denotes the nominal rejection threshold. Counts at α = 10⁻⁵ were sparse and are not used as the primary calibration evidence.

## Supplementary Table S4. Aggregate power summary

| **Method** | **Conditions** | **Mean power** | **Median power** |
| --- | --- | --- | --- |
| TorchGWAS float64 | 480 | 0.8758 | 1.000 |
| Direct full OLS | 480 | 0.8758 | 1.000 |
| PLINK 2.0 OLS | 480 | 0.8758 | 1.000 |

Power was evaluated at P < 5 × 10⁻⁸ with 20 replicates per condition.

## Supplementary Table S5. Full-genome native-format end-to-end benchmark

| **Input** | **Median (s)** | **Range (s)** | **Peak host RSS (GB)** | **Peak GPU alloc./reserved (GB)** | **Output (GB)** |
| --- | --- | --- | --- | --- | --- |
| BED | 28.46 | 28.18–28.75 | 5.00 | 0.90 / 1.37 | 36.7 |
| Hard-call PGEN | 29.13 | 28.59–29.68 | 9.89 | 2.47 / 3.16 | 36.7 |
| BGEN | 51.48 | 51.10–51.87 | 6.55 | 5.35 / 8.34 | 36.7 |
| Dosage PGEN | 58.95 | 56.28–61.62 | 38.67 | 19.84 / 20.54 | 36.7 |

H100 80 GB. 48-core Intel Xeon Gold 6442Y host. N = 35,365, M = 8,931,083 and K = 512. Values are medians and ranges from two interleaved cold-cache rounds and include durable β+t output.

## Supplementary Table S6. Measured configurations shown in Fig. 2B and C

| **Comparator** | **Samples, N** | **Variants, M** | **TorchGWAS median (s)** | **Comparator median (s)** | **Faster method** |
| --- | --- | --- | --- | --- | --- |
| PLINK 2.0 OLS | 8,192 | 1,500,000 | 9.400 | 6.930 | PLINK 2.0 OLS |
| PLINK 2.0 OLS | 8,192 | 3,000,000 | 9.545 | 13.950 | TorchGWAS |
| PLINK 2.0 OLS | 32,768 | 166,400 | 4.420 | 4.025 | PLINK 2.0 OLS |
| PLINK 2.0 OLS | 32,768 | 260,000 | 4.895 | 5.580 | TorchGWAS |
| fastGWA-lr | 2,048 | 237,824 | 3.825 | 3.020 | fastGWA-lr |
| fastGWA-lr | 2,048 | 543,744 | 4.273 | 6.833 | TorchGWAS |
| fastGWA-lr | 8,192 | 65,024 | 3.221 | 2.849 | fastGWA-lr |
| fastGWA-lr | 8,192 | 130,048 | 3.570 | 6.478 | TorchGWAS |
| fastGWA-lr | 32,768 | 10,240 | 3.731 | 2.922 | fastGWA-lr |
| fastGWA-lr | 32,768 | 15,616 | 3.946 | 4.099 | TorchGWAS |

Rows report the measured configurations plotted in Fig. 2B and C. Each reported central value is the median of two fresh analytical invocations for $K=1$on the H100 system, using the timing boundary defined in Section S4.1 and ending after native output completion. PLINK 2.0 used 48 requested threads and page-cache-preconditioned PGEN input. The fastGWA-lr comparisons used matched runs after input-page eviction. Within each sample size, the displayed points lie on opposite sides of the predicted crossover. The predicted M values were 422,911, 103,419 and 13,273 for fastGWA-lr at N=2,048, 8,192 and 32,768, and 1,545,216 and 236,542 for PLINK 2.0 at N=8,192 and 32,768. All five predictions fall inside their corresponding measured brackets. Predicted crossover boundaries were not fitted to these points and remain approximate.

# Supplementary Figures

**Supplementary Figure S1**. Numerical concordance of TorchGWAS with direct full OLS. Panels compare β, standard error, t statistic and two-sided P value across 192 variant–phenotype tests with three genotype PCs as covariates. Direct full OLS is on the x-axis and TorchGWAS float64 is on the y-axis. The diagonal denotes equality. The P-value panel uses logarithmic axes.

**Supplementary Figure S2**. Structured-population null calibration. Panels show observed-to-expected type I error, genomic inflation and representative QQ plots across increasing proportions of phenotype variance explained by genotype PCs. TorchGWAS and PLINK 2.0 overlap. Omitting PCs produces inflation and the original phenotype-only formula is mildly conservative.

**Supplementary Figure S3**. Structured-population power in a representative strong-structure setting. Power at P < 5 × 10⁻⁸ is plotted against positive effect size for one causal variant, 20% PC-explained phenotype variance and strong causal-variant–PC correlation. Panels show realized MAF groups near 0.01, 0.05, 0.20 and 0.40. TorchGWAS, direct full OLS and PLINK 2.0 overlap. Points are estimates from 20 replicates and error bars are exact 95% binomial confidence intervals.
